# Synthesis and Characterization of Coordinatively Unsaturated Copper (II) Complexes of 1,3-Bis(2'-Pyridyl)-1,2-Diaza-2-Butene and Their Antityrosinase Activity

**DOI:** 10.1155/MBD.1998.59

**Published:** 1998

**Authors:** Ratnamala Bendre, Anupa Murugkar, Subhash Padhye, Pallavi Kulkarni, Meena Karve

**Affiliations:** Department of Chemistry and Biochemistry University of Pune Pune 411 007 India

## Abstract

The coordinatively unsaturated copper (II) complexes of 1,3-bis(2'-pyridyl)-1,2-diaza-2- butene with different ancillary anions were synthesized which can bind to copper centers of tyrosinase enzyme. The compounds were found to exhibit inhibitory activities against mushroom tyrosinase and the nature and extent of inhibition is modulated according to the type of ancillary anions.


### SYNTHESIS AND CHARACTERIZATION OF COORDINATIVELY UNSATURATED COPPER (II) COMPLEXES OF 1,3-BIS(2'-PYRIDYL)-1,2-DIAZA-2-BUTENE AND THEIR ANTITYROSINASE ACTIVITY

Ratnamala Bendre, Anupa Murugkar, Subhash Padhye*, Pallavi Kulkarni and Meena Karve

Department of Chemistry and Biochemistry, University of Pune, Pune-411 007, India

### Abstract

The coordinatively unsaturated copper (II) complexes of 1,3-bis(2'-pyridyl)-1,2-diaza-2-butene with different ancillary anions were synthesized which can bind to copper centers of tyrosinase enzyme. The compounds were found to exhibit inhibitory activities against mushroom tyrosinase and the nature and extent of inhibition is modulated according to the type of ancillary anions.

### Introduction

The observation of Prabhakaran and Kircheimer [1] regarding *Mycobacterium leprae* being unique among mycobacteria in possessing a Dopa (dihydroxyphenylalanine) uptake and tyrosinase (3,4-diphenoloxidase) enzyme system has remained controversial while some ascribing it as simple amino acid uptake of no special significance [2] and others ascribing the dopa-dopaquinone system as part of the possible respiratory chain [3].The intracellular accumulation of radiolabelled pigments following incubation with $^{14}$C-Dopa, however, indicates some selective oxidative process operative in the organism [4]. The role of such an enzyme-uptake-respiratory system, although, is still not confirmed, this putative enzyme provides a possible selective target for the design of novel antileprotic compounds which would possess altogether different structures from the conventional antileprotic drugs [5].

Based on this approach a series of indole analogues having general structure (**1**) were designed as possible inhibitors of tyrosinase [6] while in another study substituted aromatic and heterocyclic carboxylic acids were tested as putative inhibitors against mushroom tyrosinase as a model system [7].

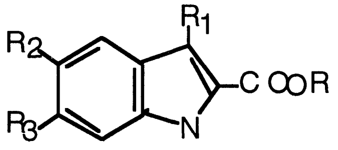

(**1**)

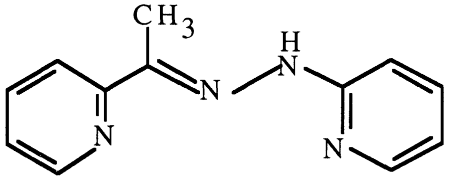

(**2**)

The fact that the successful inhibitors in this model system show good antileprotic activity *in vitro* when tested against *M. leprae* [5] motivated us to consider it as a preliminary rapid screen for evaluating further putative compounds belonging to imidazole series of ligands and their metal complexes.

The present work deals with the synthesis and characterization of three water soluble copper complexes of the ligand 1,3-bis(2'-pyridyl)-1,2-diaza-2-butene (**PPHY, 2**), which is a neutral, tridentate Schiff base derivative obtained by the condensation reaction of 2-

acetylpyridine with 2-hydrazinopyridine, and their tyrosinase inhibitory activities. Analogous heterocyclic hydrazone derivatives have been proposed as acceptable replacements for the active but cytotoxic thiosemicarbazone antileprotic compounds. These compounds have been found to be effective against *M. lufu* independently or in combination with dapsone or trimethoprim [8]. Our studies indicate that the copper compounds of **PPHY** exhibit inhibitory activities against mushroom tyrosinase and the nature of their inhibition can be modulated according to the nature of ancillary anions.

### Materials and Methods :

### Chemicals and Instrumentation :

All solvents and reagents used were of AR grade. 2- acetylpyridine was obtained from Aldrich while 2-hydrazinopyridine, L-DOPA (L-3,4-dihydroxyphenylalanine) and mushroom tyrosinase (EC 1.14.18.1) were products of Sigma Chemicals Company.

The elemental analyses were carried out in the Microanalytical Laboratory of University of Pune. The conductance measurements were made on a Phillips GM 4144 Conductivity bridge. Magnetic susceptibilities of the complexes were determined at 300 K on a Faraday type magnetic balance with a permanent magnet having field of 7000 KG. Absorption spectra were recorded as nujol mulls on the Shimadzu UV-160 spectrophotometer. Infrared Spectra were recorded as nujol mulls on a PYE-UNICAM 9512 IR spectrometer. ESR spectra of the polycrystalline samples were recorded on a Varian 109 ESR spectrophotometer at 300K with 100 KHz field modulation amplitude.

The enzyme inhibitory studies were made using L-dopa as the substrate following literature protocols [9].

### Preparation of 1,3-bis(2'-pyridyl)-1,2-diaza-2-butene (PPHY, 2) and its metal complexes :

**PPHY** was synthesized by refluxing 5 ml of ethanolic solution of 2-acetylpyridine (0.005 mol) with an ethanolic solution of 2-hydrazinopyridine (0.005 mol in 10 ml ethanol) for 10 minutes. A slow addition of water to the cooled reaction mixture separated out the ligand **2 (PPHY)** as a white solid which was filtered, washed with cold ethanol and diethylether and dried under vacuum.

For the preparation of the complex [Cu(PPHY)$Cl_2$], a hot ethanolic solution of $CuCl_2.2H_2O$ (0.002 M) was added to the hot solution of **PPHY** (0.002 M ) in *ca.* 15 ml ethanol. The resulting solution was refluxed for 10 to 15 minutes and cooled to room temperature when a green product (**A**) separated out which was filtered, washed with small portions of cold ethanol and ether and dried in vacuum. Other complexes, viz. [Cu(PPHY)Cl]$ClO_4$ (**B**) and [Cu(PPHY)$ClO_4$]$ClO_4$ (**C**), were synthesized by the addition of stoichiometric amounts of solid $NaClO_4$ to the ethanolic reaction mixtures containing metal salts and the ligand. The resulting products were filtered, washed with cold ethanol and ether and dried in vacuum.

### Results and Discussion

The synthesized copper complexes are shades of yellow to green and are stable at room temperature. They are readily soluble in water and other organic polar solvents. The compounds are coordinatively unsaturated as indicated by the conductivity data in ethanol which indicate them to have a metal to ligand ratio of 1:1 with a general formula of [M(HL)X)]Y, where HL is the neutral ligand and X and Y are the ancillary anions. All of the synthesized complexes exhibit a broad IR absorption around 3350 $cm^{-1}$ corresponding to the protonated hydrazino group which confirms the neutral nature of the hydrazone ligand in these compounds. In the IR spectra of the ligand **2**, a sharp band observed at 1600 $cm^{-1}$ is ascribable to the imino (C=N) stretch which is found to undergo a shift to the higher wavenumbers upon complexation with copper ions (**Table 1**). Such upward shifts of the imino functionality have been interpreted as indication of its involvement in the metal coordination [10]. Similar upward shifts can also be noted for the medium intensity band near 1000 $cm^{-1}$ ascribable to the N-N stretching frequency.

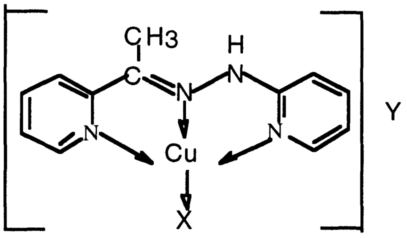

Complex **A** & **C**

**A** : X=Y=Cl

**C** : X= Y= ClO4

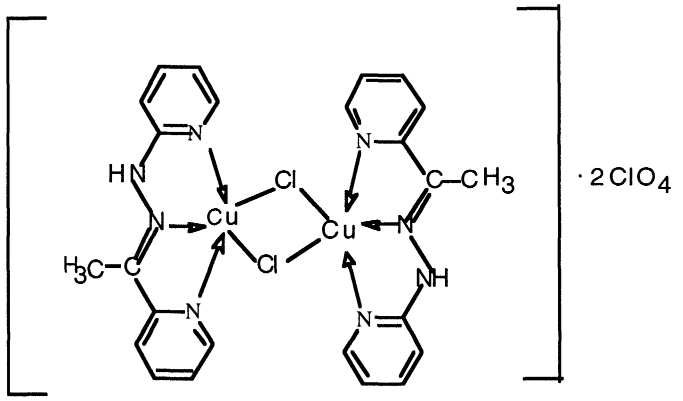

Complex **B**

Five sharp and characteristic pyridine stretches can be observed for the ligand as well as their copper compounds between 1350 and 1600 $cm^{-1}$ similar to the metal complexes of an analogous ligand, viz. pyridine-2-carboxaldehyde-2-pyridyl hydrazone [11]. It has been established that the complexes of the hydrazino ligands behave much like the complexes of the pyridine ligands [12]. Finally, the perchlorate anions have been shown to exhibit two bands in the IR spectra at 1095 and 620 $cm^{-1}$ corresponding to asymmetric stretching and bending frequencies respectively [13].These can be seen in the present **B** and **C** complexes around 1090-1095 and 628-630 $cm^{-1}$ respectively which confirm the presence of anionic perchlorate ions associated with these compounds. A low energy band observed in the dimeric complex **B** around 310 $cm^{-1}$ may be assigned to the Cu-Cl bridging mode [14].

**Table 1.** Spectroscopic, magnetic, EPR and conductivity data of Cu(II) complexes.

| Compound | IR Spectra[a], $cm^{-1}$ | | | Electronic Spectra[a] | $\mu_{eff}$[b] | $g_{av}$[c] | M($ohm^{-1}cm^{2}mol^{-1}$)[d] |
|---|---|---|---|---|---|---|---|
| | ν(N-H) | ν(C=N) | ν(N-N) | $\lambda_{max}$ ($cm^{-1}$) | (BM) | | |
| PPHY | 3350 | 1600 | 1000 | - | - | - | - |
| A | 3305 | 1620 | 1025 | 14124, 20000 | 1.74 | 2.12 | 11 |
| B | 3280 | 1640 | 1020 | 14926, 22548 | 1.32 | 2.10 | 47 |
| C | 3250 | 1625 | 1020 | 14388, 20691 | 1.76 | 2.08 | 42 |

[a] In nujol mull; [b] At 300 K, [c] Powder X-band spectra at 300 K; [d] In ethanol

The compounds show a broad band at *ca.* 14000 $cm^{-1}$ probably as the result of the overlapping of two d-d transitions and a shoulder around 20,000 $cm^{-1}$ respectively. Additionally ligand to metal charge transfer band can be seen around 25000 $cm^{-1}$ while the intra-ligand $n \rightarrow \pi^*$ bands involving azomethine nitrogen lone pair are observed at ca. 29000 $cm^{-1}$. The latter are found to undergo a shift to the lower energy side upon metal complexation.

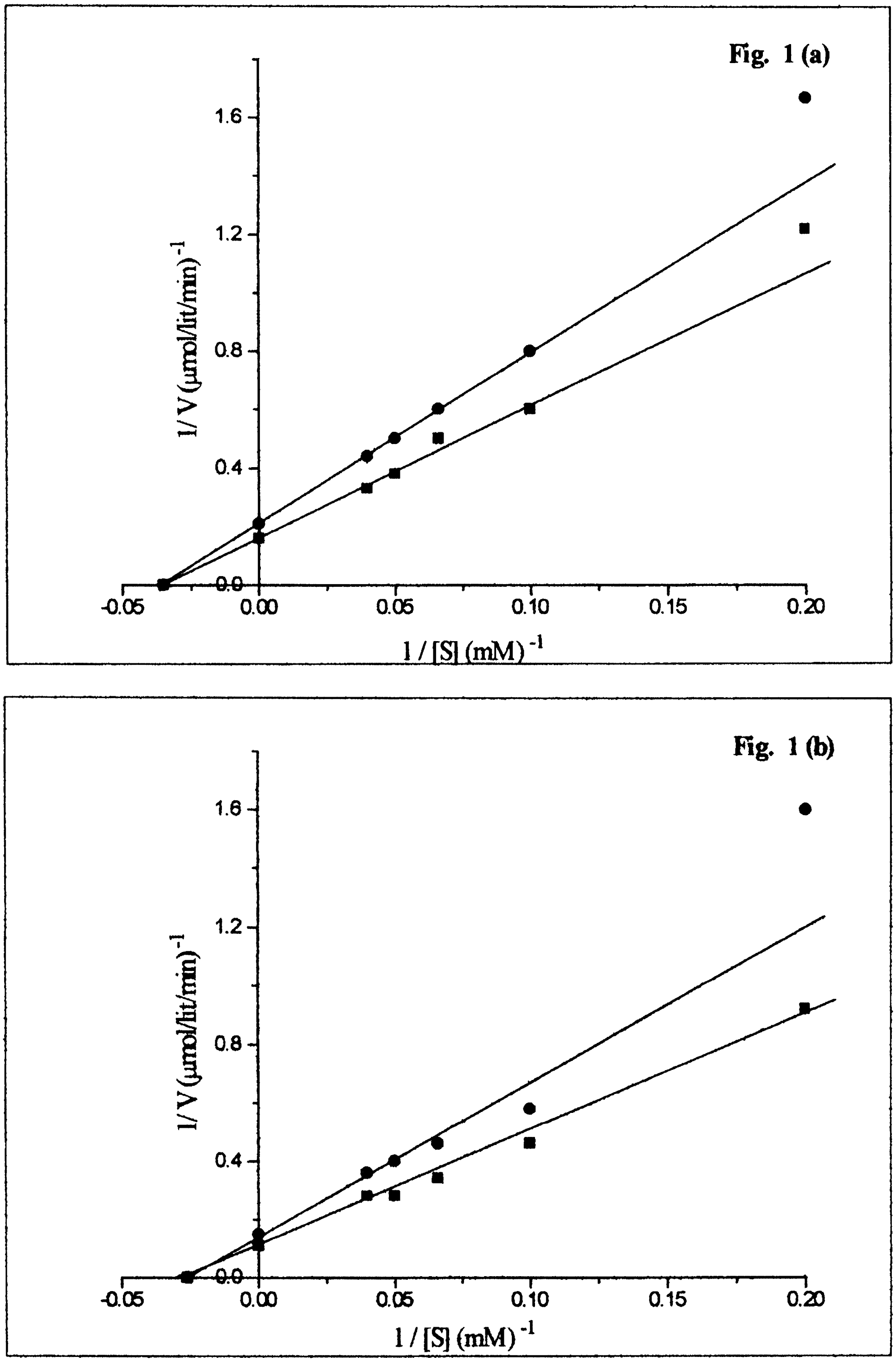

**Figure 1**: Double reciprocal plots showing competitive inhibition of tyrosinase (a) thiourea and (b) **PPHY** •With 1.0 x $10^{-4}$M inhibitor concentration ■ Without inhibitor

**Table 2.** Inhibitory effects of copper complexes on Mushroom Tyrosinase activity

| Inhibitor | Concentration (M) | Ancillary Ligands | % Inhibition | $K_i$ (M) | Type of Inhibition |
|---|---|---|---|---|---|
| Thiourea | $1.0 \times 10^{-2}$ | - | 28% | $2.6 \times 10^{-2}$ | NC |
| PPHY | $5.0 \times 10^{-4}$ | - | 13% | $3.6 \times 10^{-3}$ | NC |
| A | $1.0 \times 10^{-4}$ | Cl, Cl | 10% | $1.0 \times 10^{-3}$ | NC |
| B | $1.0 \times 10^{-4}$ | Cl, $ClO_4$ | 11% | $4.5 \times 10^{-4}$ | C |
| C | $1.0 \times 10^{-4}$ | $ClO_4$,$ClO_4$ | 25% | $1.4 \times 10^{-4}$ | UC |

NC - Non-competitive; C - Competitive; UC - Uncompetitive

ESR spectra of the present complexes are typical of compounds having an axial geometry with $g_{\|} > g_{\perp}$. The evidence for the dimeric nature of the complex **B** is provided by the weak multiline features observed around 1600 G corresponding to the MS = $\pm 2$ forbidden transition. The G value calculated from the relation $G = (g_{\|}-2)/(g_{\perp}-2)$ is found to be close to 4.0 which indicates weak magnetic exchange interactions between the two copper centers of complex **B.** This is further supported by the lowered magnetic moment (1.32 at 300 K) of the compound **B** which is ascribable to the antiferromagnetic interactions through the superexchanges [15].Comparison of the g values ($g_{\|}>g_{\perp}>2.03$) suggests the presence of the unpaired electron in the $d_{x^2-y^2}$ orbital and an approximate $D_4h$ symmetry for the present compounds. Similar configurations have been proposed for the copper complexes of analogous NNN donor ligands like TPT (2,4,6-tris(2-pyridyl)-1,3,5- triaxine) [16] BPCA (N-2-pyridyl carbonyl -2-pyridine carbiximidate) [17] .

### Tyrosinase Inhibitory Activity

The tyrosinase inhibitory activities of the three copper complexes were measured spectrophotometrically in a phosphate buffer (0.1 M, pH 6.0) at 475 nm alongwith the parent ligand (**PPHY**) and thiourea as the standard non-competitive inhibitor [18]. The results are summarized in **Table 2. PPHY** showed 13 % non-competitive inhibition at a concentration of $5 \times 10^{-4}$ M similar to thiourea which showed a non-competitive 28 % inhibition (**Fig.1**).

It is observed that complexation of copper ions with different ancillary anions seem to influence not only the extent of inhibition (from 10 to 25 %) but also the nature of inhibition from non-competitive to competitive one (**Fig. 2 a-c**) suggesting that binding of these coordinatively unsaturated complexes at the active site of the enzyme is perhaps influenced by the nature and bulk of ancillary ligands. This observation is important as it not only establishes the usefulness of copper complexes as tyrosinase inhibitors but also indicates that their antityrosinase activity can be modulated through choice of appropriate ancillary anions.

**Acknowledgements** : RB and AM thank CSIR for Senior Research Fellowship. SP and MK gratefully acknowledge the support from AICTE and British Council.

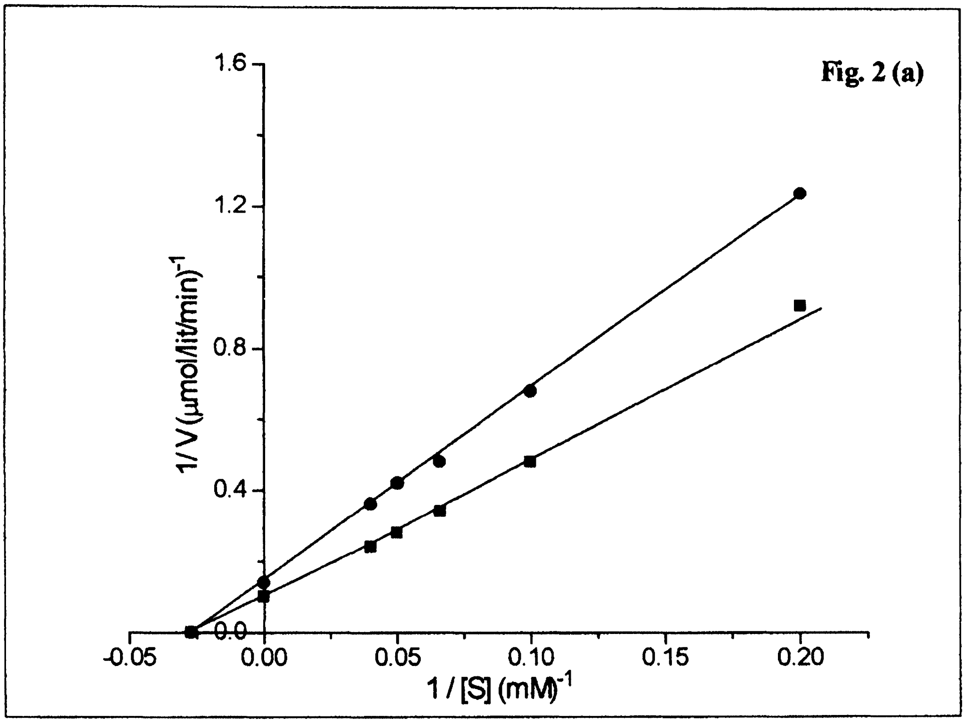

Fig. 2 (a)

1/V (μmol/lit/min)-1

1.6

1.2

0.8

0.4

0.0

-0.05

0.00

0.05

0.10

0.15

0.20

1 / [S] (mM)-1

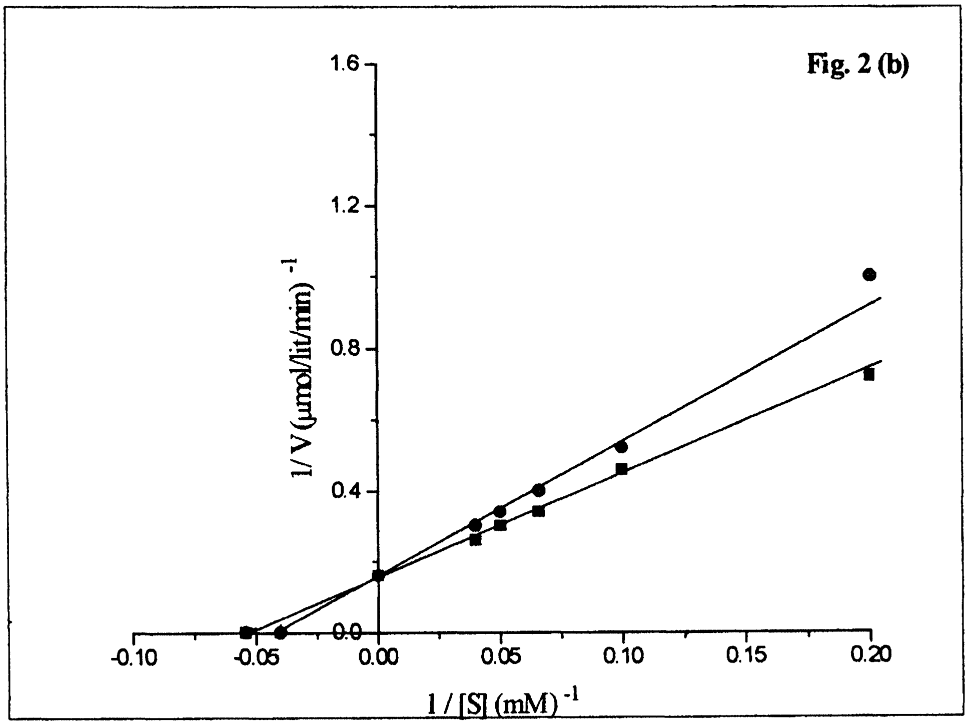

Fig. 2 (b)

1/V (μmol/lit/min) -1

1.6

1.2

0.8

0.4

0.0

-0.10

-0.05

0.00

0.05

0.10

0.15

0.20

1 / [S] (mM) -1

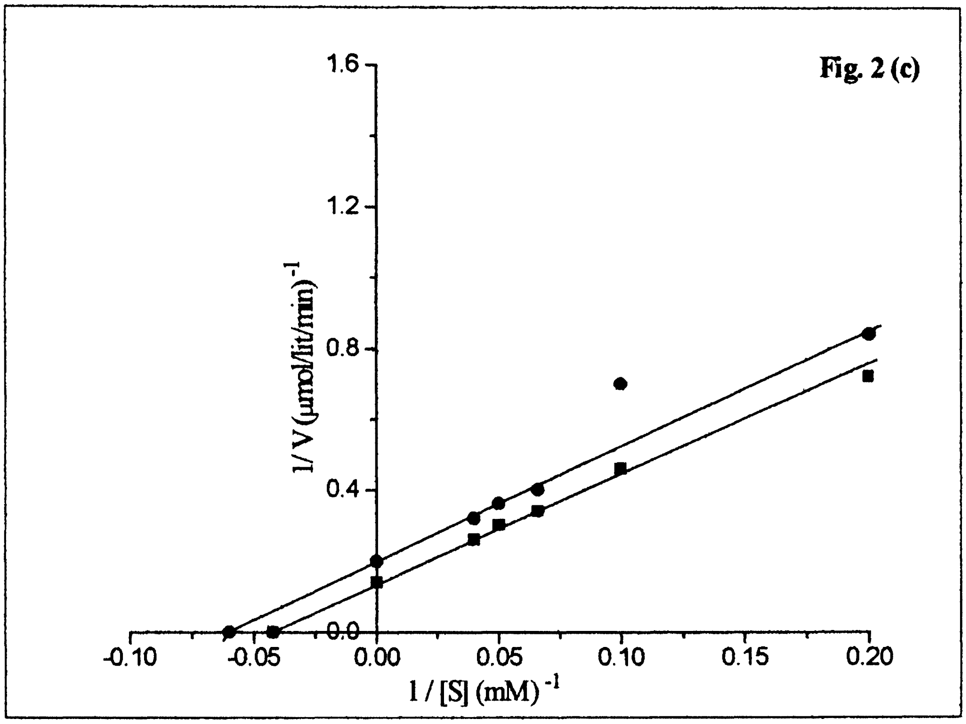

**Figure 2**: Double reciprocal plots showing noncompetitive, competitive and uncompetitive inhibition of tyrosinase by (a) Complex **A**, (b) Complex **B** & (c) Complex **C**
• With 1 X $10^{-4}$ M inhibitor concentration ■ Without inhibitor

### REFERENCES :

1. Prabhakaran K., Kircheimer W.F., *J.Bacteriol.*, **92**, 1267-1268(1966).
2. Wheeler P.R., *Int.J.Lepr.*,**52**, 208-230 (1984).
3. Robinson E.S., Nelson J.M., *Arch.Biochem.*,**4**, 111-116 (1994).
4. Ambrose E.J., Antia N.H., Khanolkar S.R., *Nature (London)*,**249**, 854-855 (1974).
5. Hooper M., Purohit M.G., *Prog.Med.Chem.* (Ed. Ellis G.P, West G.B, Elsevier) **20**,1-69(1983).
6. Yeap S.K., *Diss.Abstr.Int.B*, **49**, 84 (1987) .
7. Beveridge A., *J.Pharm.Pharmacol.*, **37**, 149 (1987) .
8. Schaper K.J., Seydel J.K., Rosenfeld M., Kazda J., *Lepr.Rev.*,**57**, 254-264 (1986).
9. Fling M.,Horowitz N.H., Heinemann S.F., *J.Biol.Chem.*, **238**, 2045-2053 (1963).
10. Burger K., In *Coordination Chemistry: Experimental Methods*, Translated by Bogyo G; Millar IT, Allen DW,eds. Butterworths,London; 94-95 (1973).
11. Mihkelson A.E., *J.Inorg.Nucl.Chem.*, **43**, 123-126 (1981).
12. Gill N.S., Nuttal R.H., Scaife D.E., Sharp D.W.A., *J.Inorg.Nucl.Chem.*,**18**,79-87 (1961).
13. Wickenden A.E., Krause R.A., *Inorg.Chem.*, **4**, 404-407 (1965).
14. Smith D.W., *Coord.Chem.Rev.*, **21**, 93-158 (1976).
15. Barefield E.K., Busch D.H., Nelson S.M., *Quart.Revs.*, 457-498 (1968).
16. Folgado J.V., Escriva E., Beltran-Porter A., Beltran-Porter D.,*Transition Met.Chem.*, **11**, 485-488 (1986).

17. Folgado J.V., Escriva E., Beltran-Porter A., Beltran-Porter D., *Transition Met.Chem.*, **12**, 306-310 (1987).
18. Duckworth H.W., Coleman J.F., *J.Biol.Chem.*, **245**, 1613-1625 (1970).

**Received: November 11, 1997 - Accepted: December 3, 1997 - Received in revised camera-ready format: January 26, 1998**

